# A randomized controlled trial to evaluate the Make Safe Happen® app—a mobile technology-based safety behavior change intervention for increasing parents’ safety knowledge and actions

**DOI:** 10.1186/s40621-018-0133-3

**Published:** 2018-03-12

**Authors:** Lara B. McKenzie, Kristin J. Roberts, Roxanne Clark, Rebecca McAdams, Mahmoud Abdel-Rasoul, Elizabeth G. Klein, Sarah A. Keim, Orie Kristel, Alison Szymanski, Christopher G. Cotton, Wendy C. Shields

**Affiliations:** 1 Center for Injury Research and Policy, Research Institute at Nationwide Children’s Hospital, 700 Children’s Drive, Research Building 3, WB5409, Columbus, OH 43205 USA; 2 College of Medicine, Department of Pediatrics, The Ohio State University, Columbus, OH USA; 3 Center for Biostatistics, The Ohio State University, Columbus, OH USA; 4 College of Public Health, Division of Health Behavior and Health Promotion, The Ohio State University, Columbus, OH USA; 5 Center for Biobehavioral Health, Research Institute at Nationwide Children’s Hospital, Columbus, OH USA; 6 Illuminology, Columbus, OH USA; 7 Nationwide®, Columbus, OH USA; 8 Health Policy and Management, Johns Hopkins Bloomberg School of Public Health, Baltimore, MD USA

**Keywords:** Multiple injury, Behavior change, Randomized trial, Child, Home

## Abstract

**Background:**

Many unintentional injuries that occur in and around the home can be prevented through the use of safety equipment and by consistently following existing safety recommendations. Unfortunately, uptake of these safety behaviors is unacceptably low. This paper describes the design of the *Make Safe Happen®* smartphone application evaluation study, which aims to evaluate a mobile technology-based safety behavior change intervention on parents’ safety knowledge and actions.

**Methods:**

*Make Safe Happen®* app evaluation study is a randomized controlled trial. Participants will be parents of children aged 0–12 years who are recruited from national consumer online survey panels. Parents will complete a pretest survey, and will be randomized to receive the *Make Safe Happen®* app or a non-injury-related app, and then complete a posttest follow-up survey after 1 week. Primary outcomes are: (1) safety knowledge; (2) safety behaviors; (3) safety device acquisition and use, and (4) behavioral intention to take safety actions.

**Results:**

Anticipated study results are presented.

**Conclusions:**

Wide-reaching interventions, to reach substantial parent and caregiver audiences, to effectively reduce childhood injuries are needed. This study will contribute to the evidence-base about how to increase safety knowledge and actions to prevent home-related injuries in children.

**Trial registration number:**

NCT02751203; Pre-results.

**Electronic supplementary material:**

The online version of this article (10.1186/s40621-018-0133-3) contains supplementary material, which is available to authorized users.

## Background

Unintentional injury is the leading cause of death for children in the United States (U.S.) and is responsible for more deaths than the next three causes combined, that is, homicide, suicide and cancer (Center for Disease Control and Prevention 2012). Each year approximately 9000 children die, 250,000 children are hospitalized and more than 9000,000 children are treated in emergency departments for preventable injuries (resulting in approximately $87 billion in medical and societal costs) (Center for Disease Control and Prevention 2012). Numbers of child and adolescent unintentional injury deaths have not declined to the same extent as some diseases have, and resources directed at reducing child injury are not commensurate with the burden it imposes.

More than 50% of these injuries occur in and around the home, where children spend most of their time (Bergen et al. 2008). Common causes of fatal and nonfatal unintentional childhood injuries include: falls, fires and burns, drowning, poisoning, and suffocation (10 Leading Causes of Injury Deaths by Age Group Highlighting Unintentional Injury Deaths, United States-2014 [Internet] 2014; National Estimates of the 10 Leading Causes of Nonfatal Injuries Treated in Hospital Emergency Departments, United States-2013 [Internet] 2013). For example, in the U.S., residential fires account for nearly 90% of all fire-related deaths in children younger than 15 years (Baker and Waller 1989). almost 13,000 children are injured by TV tip-overs each year (De Roo et al. 2013) and medications are the leading causes of child poisoning (Baker and Mickalide 2012). Other home hazards include bunk beds (D’Souza et al. 2008). high chairs (Kurinsky et al. 2014), windows (Harris et al. 2011), stoves and ovens (D’Souza et al. 2009), button batteries (Sharpe et al. 2012), laundry packets (Davis et al. 2016; Valdez et al. 2014), and toys (Abraham et al. 2015)—ubiquitous products and home features present in nearly every U.S. home.

Many of these injuries in and around the home can be prevented through the use of safety equipment and by consistently following existing safety recommendations. Using known, effective countermeasures, such as smoke alarms, carbon monoxide detectors, stair gates, cabinet locks and latches, and anchoring TVs and furniture, can prevent or reduce the consequences of a child being injured (Kendrick et al. 2005).

The overall rates of these safety behaviors are unacceptably low; a 2015 survey of more than 1000 parents of children aged 12 and under found that 48% of parents surveyed had not secured TVs and furniture, 73% reported placing items like blankets, bumpers, and stuffed animals in the crib with their baby, 12.5% have left their young child alone in a bathtub for 5 min or longer, 30% of parents with a toddler report keeping medicines and cleaning products on a low shelf or unlocked cabinet, 14% report never checking their smoke alarm batteries (Report to the Nation: Protecting Children in Your Home 2015). Child and adolescent unintentional fatalities and injuries are a significant public health problem and resources directed at reducing child injury are not commensurate with the burden it poses. Parents and caregivers often have difficulty identifying hazards in their home (Gaines and Schwebel 2009), finding credible information and recommendations, and obtaining the safety products best-suited to their home. Interventions for increasing home safety behaviors which have ranged from the provision of educational materials, to health care provider counseling, safety product distribution, hands-on experiential learning provided in safety resource centers have been evaluated (with varying degrees of effectiveness) (Kendrick et al. 2014). However, wide-reaching, effective and readily available interventions may be needed to reach substantial parent and caregiver audiences.

Smartphone mobile applications are particularly promising in this area as they offer an efficient and cost-effective medium to deliver health-related programs and interventions. Offering targeted safety information on multiple topics via a single platform, combined with the ability to acquire child safety devices, may be a more efficient means to reach a large segment of the parent and caregiver population and may help reduce the aforementioned barriers. Unfortunately, few injury-related apps exist and fewer still have been systematically evaluated (Omaki et al. 2016).

The aim of the study described in this protocol is to evaluate the impact of and determine whether a mobile technology-based health behavior change intervention, the *Make Safe Happen®* app, is effective in increasing (1) safety knowledge; (2) safety actions/behaviors; and (3) safety device acquisition and use; and (4) behavioral intention (planning to take safety actions or acquire devices) for the prevention of unintentional home-related injuries, compared with no intervention.

## Methods

### Study design and setting

**Fig. 1:**
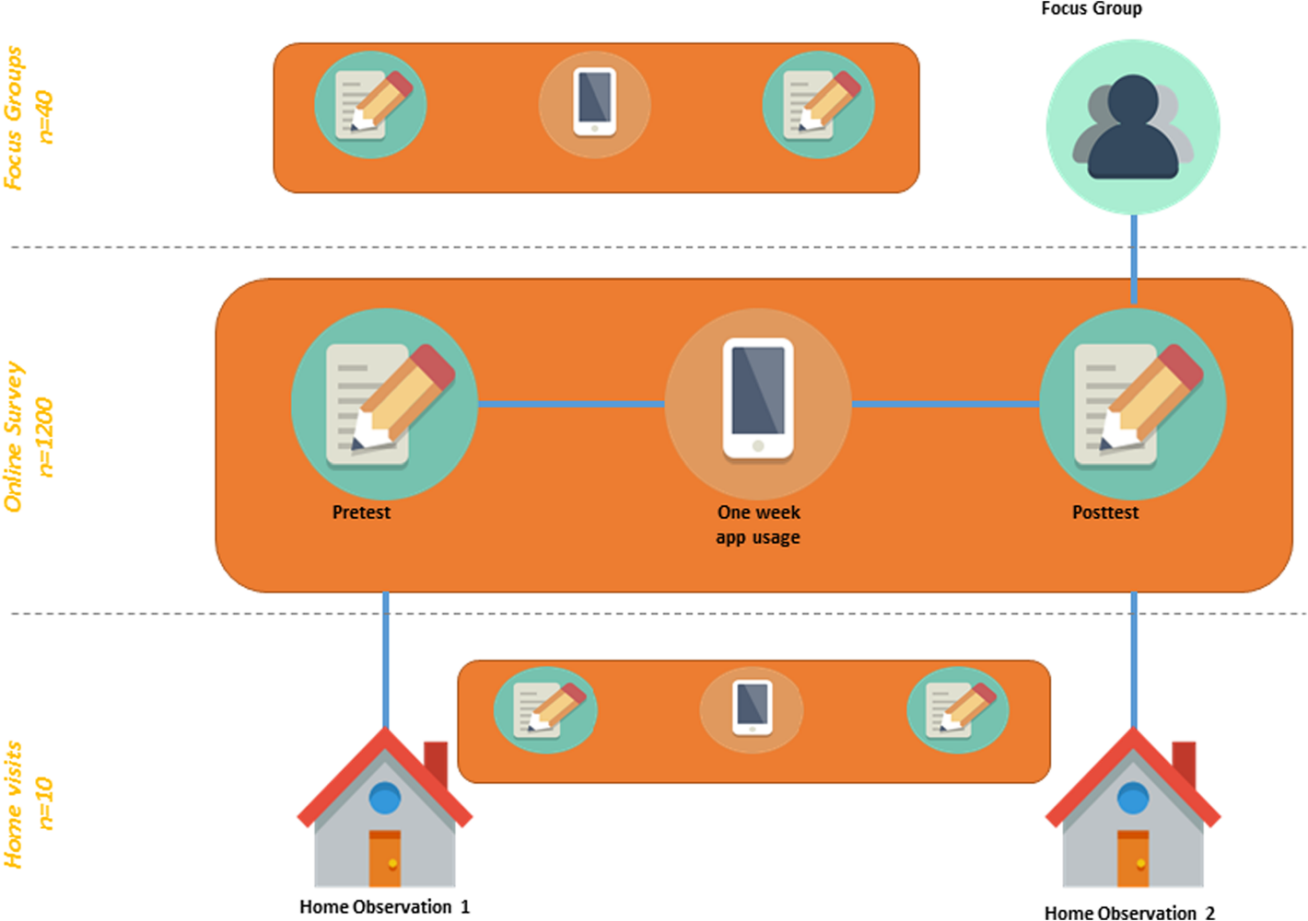
Study Design

The *Make Safe Happen®* app (Additional file 1) evaluation study will be a two-group, randomized controlled trial with pre- and posttest elements (no blinding), and will involve 1200 parents of children aged 0–12 years. These data will be collected in the U.S. The intervention group (IG) will use the *Make Safe Happen*® app and receive information about the prevention of unintentional child home-related injuries. The “attention-matched” control group (CG) will use an app to receive information about dinner recipes. Surveys measuring safety knowledge, behaviors and behavioral intentions, and safety device acquisition and use will be administered before and after app use (Fig. 1). This study was approved by the Institutional Review Board (IRB) at Nationwide Children’s Hospital. Plans for protocol modifications or for communications to relevant parties will be made to the study investigators, IRB, trial participants if necessary. Study investigators will have access to the final dataset. See Standard Protocol Items: Recommendations for Interventional Trials (SPIRIT) Checklist (Research Checklist) (Additional file 2).

### Screening, enrollment and randomization

Parents or legal guardians of at least one child aged 0–12 years will be recruited to participate in this study. The study participants will be members of a pre-existing, national online survey panel. This panel recruits people by a variety of methods, including random-digit-dial telephone calls, USPS mailings, and advertising on social networking websites. After individuals join the panel and participate in surveys, quality checks of their responses are performed. We will employ a standard practice recommended by the online survey company, that is, for participants who complete surveys too rapidly (i.e., one-third the median length of the survey response time) will be disqualified from participation in the study because of a concern that they will not be carefully processing the questions’ language and intent, nor accurately formulating their responses. These respondents will be treated as “incomplete” surveys and will not be included in the analyses.

For the *Make Safe Happen*® app evaluation study, panel members will receive an email inviting them to complete a brief survey that screens for eligibility. Inclusion criteria will require that participants: (1) have an Android phone or Apple smartphone (i.e., iOS or Android); (2) be a parent or legal guardian of a child (“index child”) aged 0–12 years; and (3) this child must live with them at least “most of the time.” Parents will be excluded if they have previously downloaded or used the *Make Safe Happen®* app or the control app. An “index child” will be selected via a least filled quota protocol, meaning a respondent will be assigned to a child age group depending on the needs, or quotas, for each age group for the entire sample and treatment group. When a new participant with more than one child between 0 and 12 years of age completes a survey, that participant will be assigned to a quota age group based on whichever has the smallest number of completed participant surveys at that time. The selection of an index child is only relevant for assigning participants to child age groups for recruitment to prevent unequal child age groups in the treatment groups. Birth order is not an issue in the proposed study.

**Fig. 2:**
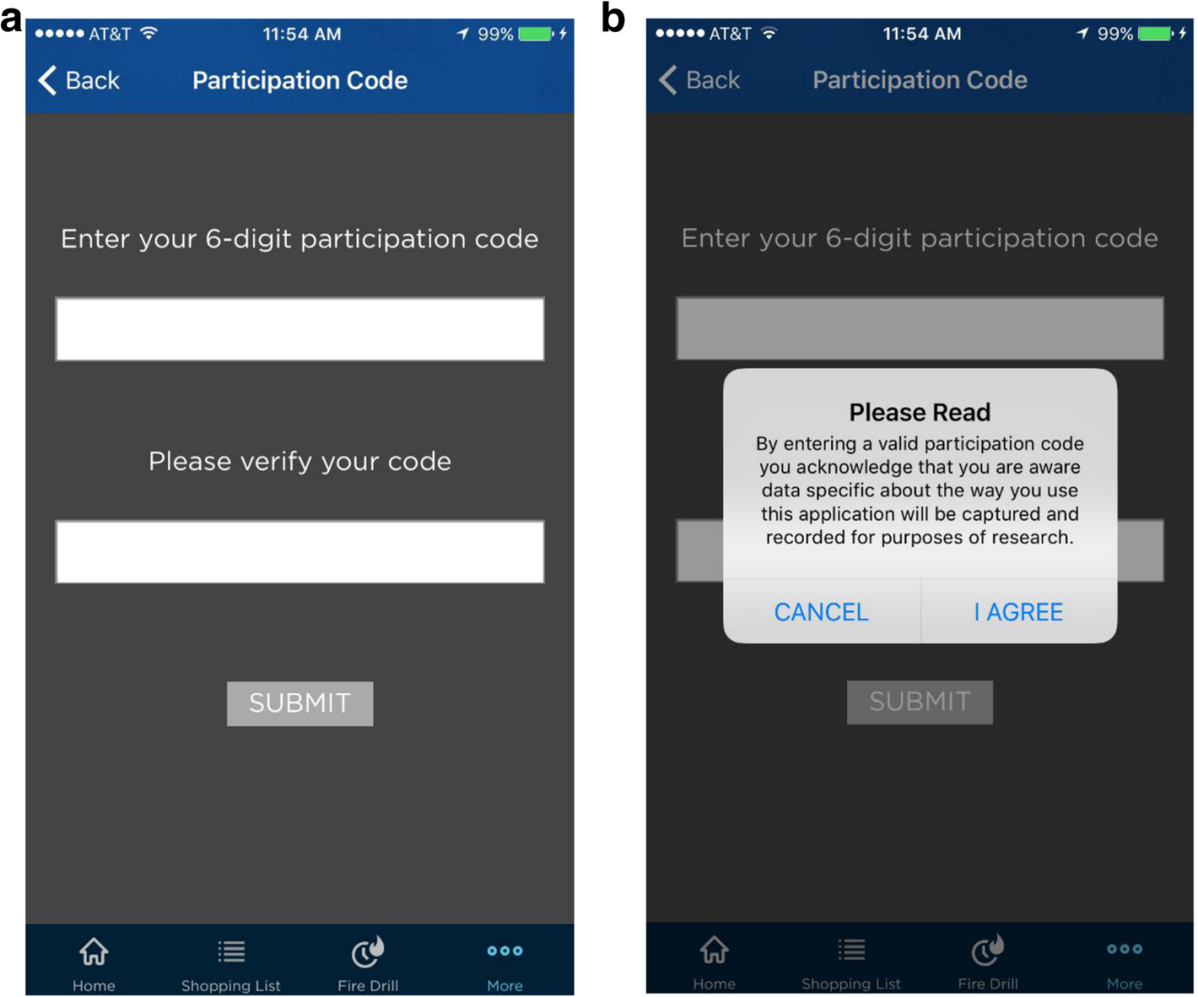
Participant Code (**a**) Participant Code Screen and (**b**) Participant Code Agreement Screen

Parents will indicate their consent to participate after reading and marking their consent to participate on an online consent form (consent language available from authors upon request). Participants will then be randomly assigned by a computer-generated roster in a 5:1 ratio to receive the *Make Safe Happen®* app Version 1.0.3 (IG) or *Allrecipes Dinner Spinner app* (CG). Randomization will be blocked in groups of five based on an index child’s age in age subgroups. After completing the pretest survey participants will be emailed and asked to download the *Make Safe Happen®* app or *Allrecipes Dinner Spinner* app from iTunes or GooglePlay app stores for free onto their smartphone. IG participants will be asked to enter a participant identification (ID) number into the *Make Safe Happen®* app which will be assigned to them after the completion of the pretest survey (Fig. 2a and b). Entering a participant ID into the *Make Safe Happen®* app will establish a link between each study participant assigned to the IG and the unique actions they take in the app over the duration of the study. This linkage will also allow participant’s app data to be connected to the participant’s pretest and posttest survey data. Participants in the IG will receive email reminders about downloading the *Make Safe Happen®* app at 24 and 72 h after they complete the pretest survey. Intervention participants who do not download the app or enter their participant ID will not be eligible to complete the posttest survey. Participants will be compensated by a point rewards system (equivalent to $8–10) when they complete all parts of the study.

### Intervention and control group content

**Fig. 3:**
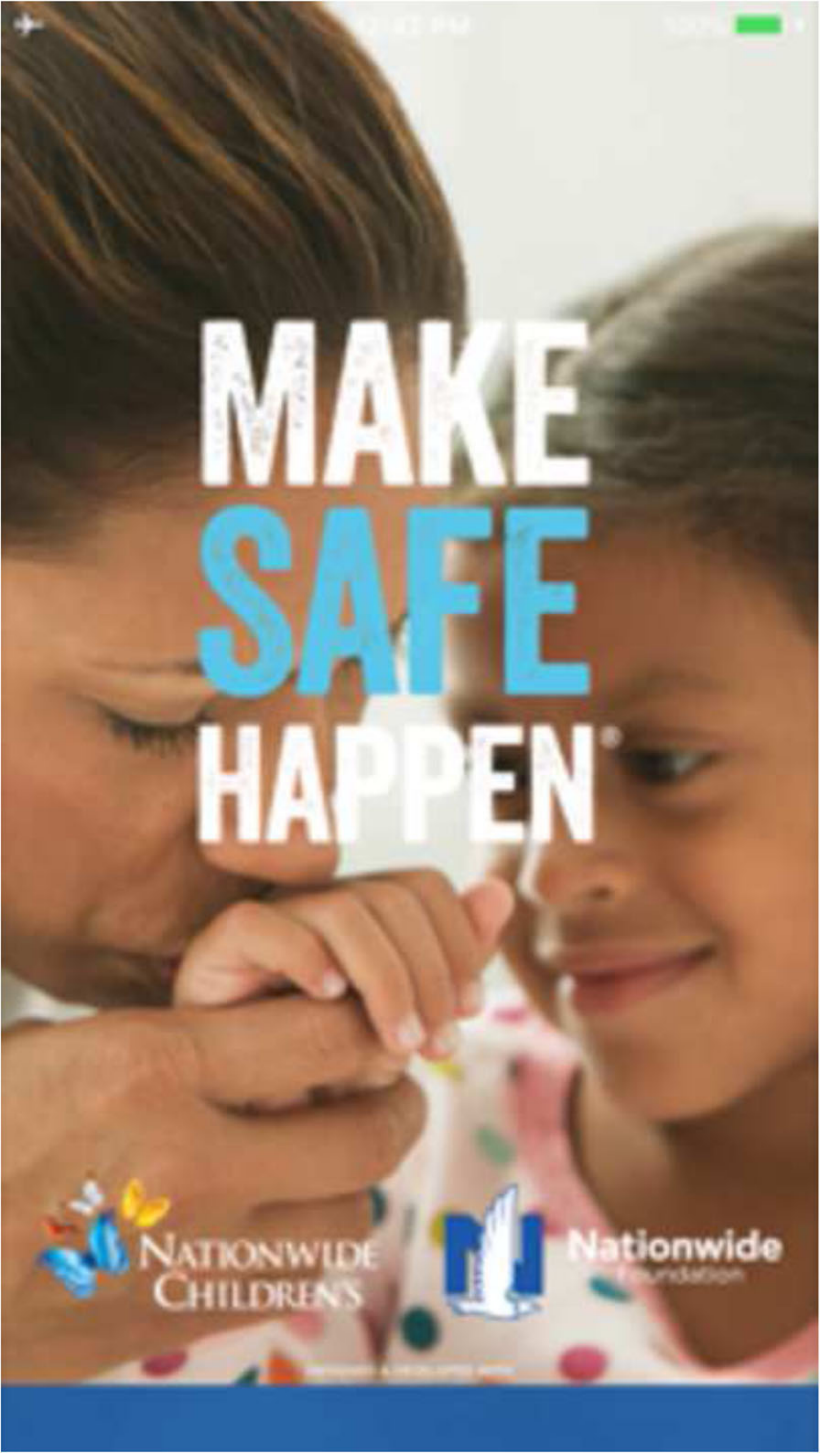
Opening Screen

**Fig. 4:**
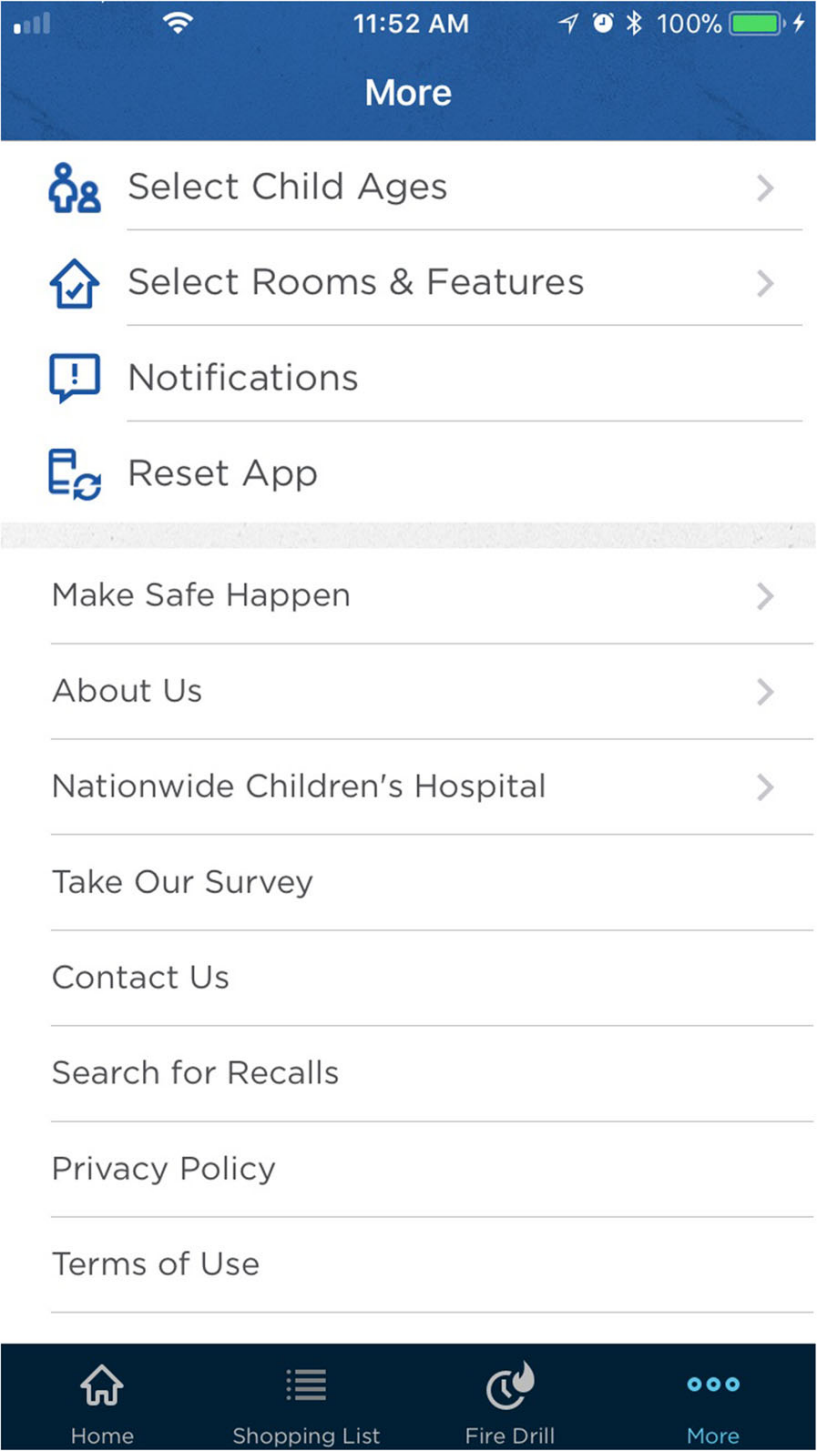
Menu Screen

**Fig. 5:**
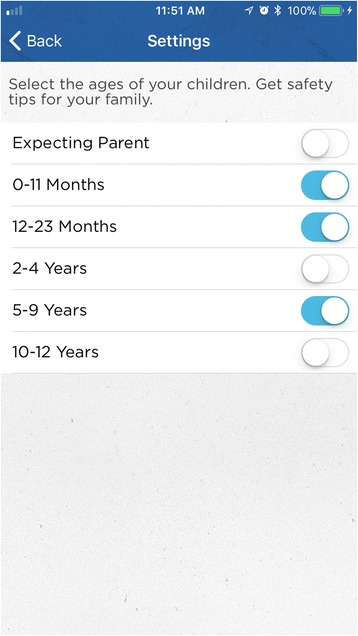
Age Selection Screen

**Fig. 6:**
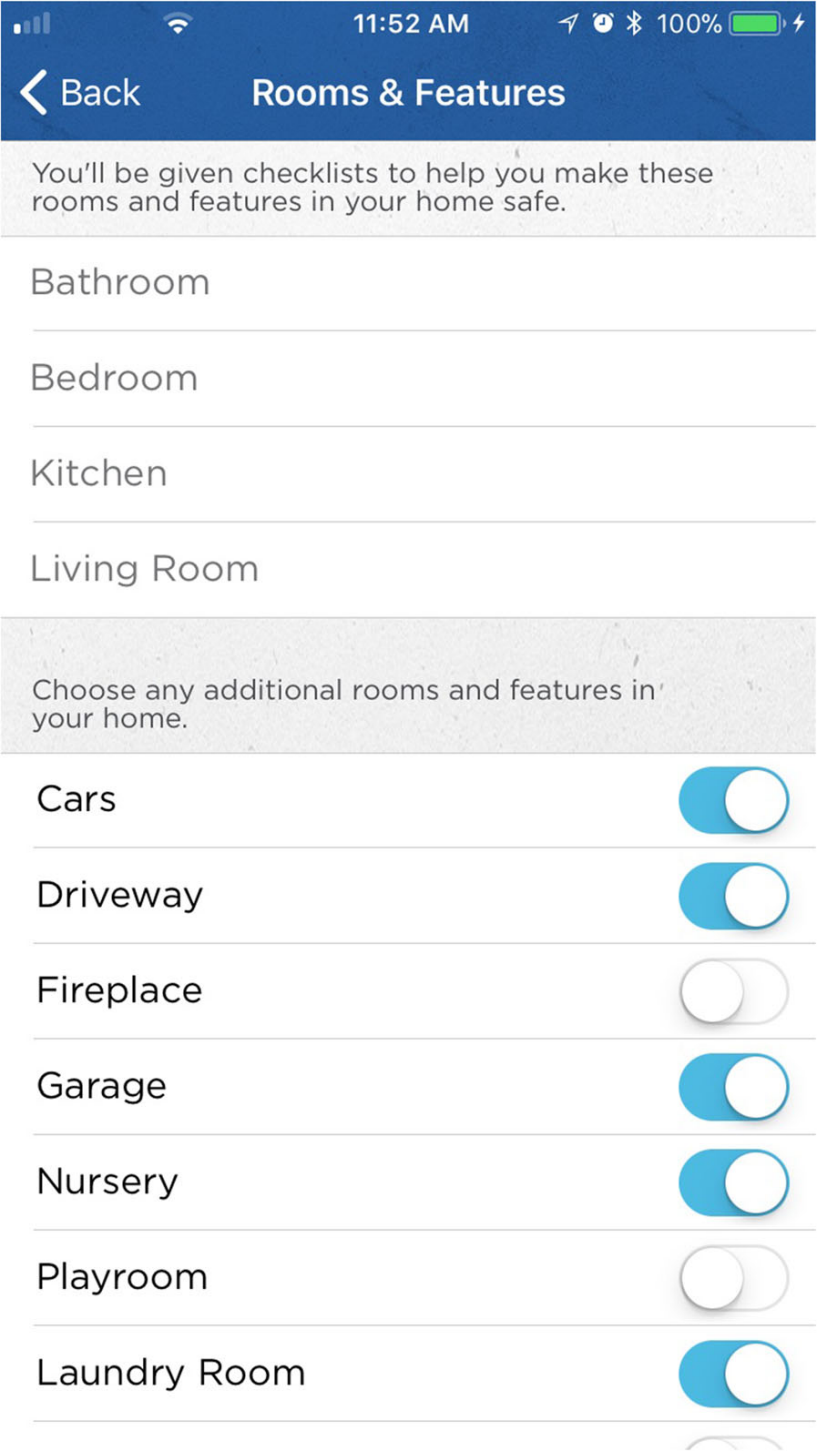
Rooms and Features

**Fig. 7:**
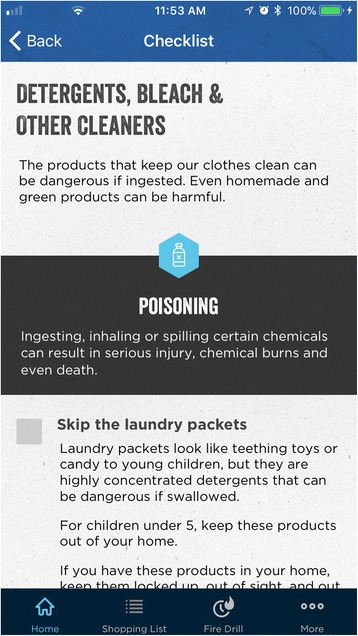
Checklist

*IG Content. Make Safe Happen® App* Version 1.0.3. In 2015, as part of a national effort, Nationwide® launched the *Make Safe Happen®* program to raise awareness of “accidental injuries” and educate parents and caregivers with tips and tools to help keep their children safe. The *Make Safe Happen®* app was created by the safety experts in the Center for Injury Research and Policy at Nationwide Children’s Hospital and developed in partnership with Nationwide® (Figs. 3 and 4). The Make Safe Happen app content and messaging was developed by utilizing principles, concepts, and constructs from the Precaution Adoption Process Model and the Theory of Reasoned Action (National Cancer Institute 2005) to reflect stage-based nature of safety action adoption and behavioral intentions associated with safety device installation and behaviors. The Make Safe Happen app presents users with “targeted communication” content (where separate audience segmentations benefit from a shared message (Rimal and Adkins 2003), which is presented as individual tasks that make up checklists by room or home feature, are presented based on the child age group(s) (these are, Expecting Parent, 0–11 months, 12–23 months, 2–4 years, 5–9 years, and 10–2 years) selected by each user. After downloading the app, and accepting the Terms of Use and Privacy Policy, users are asked to *“Select the ages of your children. Get safety tips for your family.”* General features of the app include the ability to target safety information by child’s age (Fig. 5), with room-to-room (Fig. 6) safety checklists (Fig. 7) and links to purchase common home safety products on Amazon.com. Application features also include the ability for users to create shopping lists, set reminders for testing safety devices (smoke alarms, carbon monoxide detectors) and replacing batteries, add the poison control center—emergency number to their contacts, and track their progress. Upon downloading the *Make Safe Happen®* app, all users are required to accept Privacy Policy (http://makesafehappen.com/privacy-policy) and Terms of Use (http://www.nationwidechildrens.org/safety-app-disclaimer) before selecting child ages from five age groups: 0–11 months, 12–24 months, 2–4 years, 5–9 years, and 10–12 years. Based on their age selection, home safety information presented in the app is targeted to specific selected age groups. *Make Safe Happen®* app users also have the opportunity to share the home safety content and the app via social media. Throughout the course of the intervention period and beyond, participants will have access to the *Make Safe Happen®* app. An automated notification in the app will be in effect during our study data collection period. Participants in the evaluation trial who opt in to receive notifications from the app may receive a “Welcome” notification 7 days after the app is opened for the first time. These parental engagement mechanisms will be in place, but are optional actions that users can take within the app (e.g., setting calendar reminders to take future safety actions such as testing smoke alarms or replacing batteries). The app contains links to home safety products sold on Amazon.com. Prior research by our team had identified that acquisition/purchase of safety products as a barrier to taking safety actions, however, app users are not required to purchase products from Amazon.com for the study. Further, the app contains no pop-up ads of any kind. The shopping list feature was also created so that app users could identify the safety products best-suited to the features of their home, and then create a shopping list that can be taken to their preferred store (brick and mortar or online store) to purchase. The authors and app developers do not endorse any specific brands of safety products over any others.

*CG Content. Allrecipes Dinner Spinner* (version 6.1). It is described as “the most popular food—focused social app” that helps “cooks discover and share the joy of home cooking” (App Store, Food & Drink, All Recipes, Inc. Allrecipes.com). Content will vary by user preference and choices. Download of and use of the CG content will not be confirmed by the study team. This app was selected to be used for the control condition because it does not include information about home safety and does not require that users to log in or set up an account and is available for free in iTunes and Google Play. The recipe app was selected for use by the CG participants so that they would have a similar download and app use experience to the IG group. Respondents will be asked if they downloaded Dinner Spinner when they complete the posttest survey. Participants in the CG will also receive information inviting them to download and use the *Make Safe Happen®* app after completing the posttest survey.

## Results and discussion

### Outcomes and measures

Primary outcomes will include: (1) safety knowledge; (2) safety behaviors; (3) device acquisition and use; and (4) behavioral intention to take safety actions. Participants will self-report these at baseline (pretest) and at follow-up (posttest). A combination of yes-no, multiple choice and Likert scale response options will be used. A sample of questions from the pretest and posttest surveys will be vetted for coherent meaning and level of difficulty via an hour-long cognitive interview with 20 parents prior to the launch of the study. A pilot study with 200 participants will be completed prior to the study launch to examine the distribution of participants’ responses and to address any technical difficulties with the pretest survey instrument.

#### Safety knowledge

Safety knowledge will be measured by responses to 17 questions that were created specifically for this study and are based on safety content that is delivered in the *Make Safe Happen®* app. One point will be given for each correct answer and correct responses will be summed to determine a total knowledge score for each participant. The total safety knowledge score percent will be derived by dividing the total number of correct responses by 17. The mean *total safety knowledge score* and the *mean total safety knowledge score percent* will be calculated for pretest intervention condition, pretest control condition, posttest intervention condition and posttest control condition participants.

#### Safety behaviors and behavioral intentions

Safety behaviors (either repeatedly done or one-time behaviors) and behavioral intention (planning to perform safety behaviors in the future) will be measured by responses to 31 questions. Participants will be asked about 14 safety behaviors that are recommended to repeatedly do, e.g., turning pot handles away from the stove when cooking, and 15 safety behaviors that are typically completed once, e.g., buying and installing a carbon monoxide detector. For example, participants will read the question stem, *“I repeatedly take the following safety actions in my home…”* and will be presented with several statements such as, *“Turn pot handles to the back of the stove when I cook.”* Response choices will include the following options: *always, sometimes, never, and not applicable.* Participants who report *never* doing any of these behaviors, will be asked whether they intend to in the future (*yes, no, not sure*).

Additional measures that will be collected include a count of safety actions completed (or “checked off”) within the *Make Safe Happen®* app.

#### Safety device acquisition and use

Participants will be asked whether they have any of the 22 possible types of safety devices installed in their homes, e.g., fire escape ladder, medicine lock box, etc. Response choices will include the following options: *yes, no, not sure, and not applicable.* Participants who report *no* to one or more safety devices will be asked whether they intend to purchase and install these devices in the future *(yes, no, not sure).*

### Data analysis plan

Data analysis will be primarily comprised of two activities: 1) the generation of descriptive information summarizing the sample and its characteristics; and 2) the analyses related to the aims and hypotheses. The central hypothesis is that parents in the IG will have higher safety knowledge, do more safety behaviors, have increased behavioral intention to take safety actions, and be more likely to acquire and use safety devices posttest, as compared to the CG. Linear mixed model regression will be used to analyze safety knowledge outcome and longitudinal logistic regression models will be run for behavioral intention and safety actions. Primary outcomes for the safety behaviors and device use will be assessed while stratifying on the index child age group. Analyses will be undertaken to examine the hypotheses that the IG will have higher change at posttest from baseline in safety knowledge, safety behaviors, behavioral intention, safety device use and acquisition than the CG.

Besides the intent-to-treat analyses of safety knowledge, safety behaviors and behavioral intention, safety device use and acquisition, we will also conduct exposure analyses to examine the relationship between outcomes and exposure to the intervention. For the IG participants those who enter a participant ID in the *Make Safe Happen®* app will be asked to complete a posttest survey. For the CG participants, those who complete the pretest survey will be asked to complete a posttest survey since we cannot confirm download and use of the control condition app. We plan to link app analytics for IG participants to their pretest and posttest survey responses, for example, if a participant uses the *Make Safe Happen®* app to add the poison control center emergency number to their contacts we will check to see whether that participant got the poison control center emergency number safety knowledge question correct in the pre- and posttest surveys. All study data will be stored on password protected computers of the study personnel. The study will be monitored by the study investigators.

### Sample size

We plan to enroll 5000 participants, and we estimate a 40% loss-to-follow-up, which will provide us with the needed sample size of 1200 in the posttest sample for the planned analyses. A power analysis indicated it would not be necessary to have equal numbers of participants assigned to the IG and the CG. Therefore, participants will be randomized to the treatment groups using a 5:1 ratio, allowing efficient allocation of participants to the IG or CG (respectively) within the online survey group. Focus group participants and home visit participants will not be randomized, although all focus group and home visit participants will receive the Make Safe Happen app intervention. The randomization will be completed by the use of a computer program embedded within the online survey, which randomly generates a number between 1 and 120 for each participant. Numbers between 1 and 100 will be assigned to the IG, while numbers between 101 and 120 will be assigned to the CG. Our proposed sample size reflects consideration of our proposed attrition rate and is based on previous research using online survey panels. Additionally, we will aim to recruit a diverse sample of parents ≥18 years old based on sex (30% male, 70% female), ethnicity (maximum of 66% White, non-Hispanic), and age of index child (~ 20% in each of the five age brackets).

## Discussion

Home-related unintentional injuries in children remain unacceptably high in the U.S. despite the availability of proven and effective strategies for reducing or preventing these injuries. Developing and widely disseminating effective means to communicate these strategies with parents and caregivers is a research and public health priority. This study will contribute to the evidence about how to promote the life-saving and injury-reducing benefits of home safety actions and practices.

Home safety smartphone apps are ideal for parents and caregivers. Apps can be used on parents’ own time and in the comfort of their home. Because safety recommendations are usually provided as a one-size-fits-all recommendation, parents may need information that is customized to the features in their home and to the ages of their children. Thus, this research seeks to provide home safety information, using innovative communication technology. The app can be used on their own time, in their own home, and does not rely on or involve clinicians or a medical setting and is widely available. The use of a mobile app improves upon the “one-time” nature of some brief interventions by allowing the participant or app user to revisit the app and complete safety actions as frequently as desired and to set reminders for themselves for future actions. The information provided can change as the child ages in order to be relevant for the child’s age and development, or addition of new children to the family, as well as protecting persons of all ages residing in the home. New content and features can be added in order that the *Make Safe Happen®* app can function as a virtual safety expert offering ongoing education on home safety or related topics and posts notifications about new or seasonal safety topics. Given the high rates of smartphone app use [roughly three-quarters of Americans (77%) own a smartphone] (Smith 2017),—these results should have widespread applicability.

The pretest and posttest survey data will be limited due to the nature of self-reported data and may also be subject to reporting bias. Further, the surveys will be conducted close together in time (a span of 7–10 days apart) therefore we may see a learning effect of having recently taken the survey. A follow-up period of 7 to 10 days was used in order to allow recruited participants time to download and use the app. We do not expect that participants will complete all of the recommended safety actions or safety device installations in this limited time period. We opted to keep the follow-up period relatively short in an effort to prevent loss to follow-up. While we will have actual app data to report on what users completed within the app, at best these data will be a proxy for real behaviors or safety actions. Further, the requirement for a smartphone Internet connection in order to transmit the data from the *Make Safe Happen®* app to the online platform that stores these data as well as the app data processing latency of 24—48 h may both cause delays in distribution of posttest surveys to IG participants. An additional limitation is that we will not be able to confirm or track the CG recipe app usage.

## Conclusions

This work will contribute to reducing child injury by providing an evaluation of an innovative, targeted, free and widely available smartphone app that delivers focused home safety information to parents and caregivers. With the wide availability of smartphone apps, the results of this work will have utility for disseminating behavior change programs to a broad audience, and the results of this study will advance the application of behavior change interventions in a new area—injury prevention. Further, the systematic evaluation of apps is rare in the published literature, so this protocol may help demonstrate how others (even those outside the injury prevention field) can accomplish this type of rigorous evaluation.

## Additional files

Additional file 1: Make Safe Happen app video. (MP4 112000 kb)

Additional file 2: Standard Protocol Items: Recommendations for Interventional Trials (SPIRIT) Checklist. (PDF 82 kb)

## Abbreviations

app: application
CG: Control group
IG: Intervention group
